# Elevated Systemic Levels of Eosinophil, Neutrophil, and Mast Cell Granular Proteins in *Strongyloides Stercoralis* Infection that Diminish following Treatment

**DOI:** 10.3389/fimmu.2018.00207

**Published:** 2018-02-09

**Authors:** Anuradha Rajamanickam, Saravanan Munisankar, Yukthi Bhootra, Chandra Kumar Dolla, Thomas B. Nutman, Subash Babu

**Affiliations:** ^1^National Institutes of Health – National Institute of Research in Tuberculosis (ICMR) – International Center for Excellence in Research, Chennai, India; ^2^National Institute of Research in Tuberculosis (ICMR), Chennai, India; ^3^Laboratory of Parasitic Diseases, National Institute of Allergy and Infectious Diseases, National Institutes of Health, Bethesda, MD, United States

**Keywords:** eosinophils, neutrophils, mast cells, granular proteins, helminths, *Strongyloides stercoralis*

## Abstract

Infection with the helminth parasite *Strongyloides stercoralis* (*Ss*) is commonly clinically asymptomatic that is often accompanied by peripheral eosinophilia. Granulocytes are activated during helminth infection and can act as immune effector cells. Plasma levels of eosinophil and neutrophil granular proteins convey an indirect measure of granulocyte degranulation and are prominently augmented in numerous helminth-infected patients. In this study, we sought to examine the levels of eosinophil, neutrophil, and mast cell activation-associated granule proteins in asymptomatic *Ss* infection and to understand their kinetics following anthelmintic therapy. To this end, we measured the plasma levels of eosinophil cationic protein, eosinophil-derived neurotoxin, eosinophil peroxidase, eosinophil major basic protein, neutrophil elastase, myeloperoxidase, neutrophil proteinase-3, mast cell tryptase, leukotriene C4, and mast cell carboxypeptidase-A3 in individuals with asymptomatic *Ss* infection or without *Ss* infection [uninfected (UN)]. We also estimated the levels of all of these analytes in infected individuals following definitive treatment of *Ss* infection. We demonstrated that those infected individuals have significantly enhanced plasma levels of eosinophil cationic protein, eosinophil-derived neurotoxin, eosinophil peroxidase, eosinophil major basic protein, elastase, myeloperoxidase, mast cell tryptase, leukotriene C4, and carboxypeptidase-A3 compared to UN individuals. Following the treatment of *Ss* infection, each of these granulocyte-associated proteins drops significantly. Our data suggest that eosinophil, neutrophil, and mast cell activation may play a role in the response to *Ss* infection.

## Introduction

*Strongyloides stercoralis (Ss)*, an intestinal parasitic nematode, infects 30–100 million people worldwide (1). The clinical manifestation of *Ss* can range from clinically asymptomatic to, at its most severe, a potentially fatal disseminated infection. Granulocytes are activated during helminth infection and act as immune effector cells. *In vitro* granulocyte mediated immunity against helminths can be attained through antibody-dependent cell-mediated cytotoxicity, and antibody attaches to the parasite’s cell surface and triggers degranulation and extrusion of toxic granule contents against the parasite (2).

In healthy people, eosinophils normally constitute only 2–5% of peripheral leukocytes. However, during active helminth infection, the eosinophils fraction in the blood can increase to more than 40% (3). Eosinophils have eosinophil-specific toxic proteins stored in their secondary granules. These include eosinophil cationic protein (ECP), eosinophil peroxidase (EPX), eosinophil-derived neurotoxin (EDN), and eosinophil major basic protein (MBP). ECP, EPX, and MBP are potent helminth toxins (4). MBP can provoke histamine release from mast cells; however, EDN and ECP can act as ribonucleases (4, 5). Experimental helminth infection studies revealed that eosinophils accumulate in the gastrointestinal tract, where it is believed that they assist to eliminate parasites (6). Interestingly, evidence suggests that there could be dissimilarities in the mechanisms of eosinophil-mediated killing among different life cycle stages of the same parasite (7).

Among granulocytes, neutrophils are effective at phagocytosis, and they can engulf and execute microorganisms by producing reactive oxygen intermediates in phagolysosomes. Conversely, helminths are very large to be phagocytosed, and as a outcome, the function of neutrophils in helminth-driven effector responses has been ignored till now (2). Neutrophils can be defensive against nematode parasites, and this has been exhibited conclusively in the *Strongyloides sp*. model (8). Like neutrophils, granulocytes are also critical in controlling *Streptococcus ratti* in mice (9). Myeloperoxidase (MPO) purified from human neutrophils is toxic to *Trichinella spiralis* and *Schistosoma mansoni* (10, 11) and functions in killing *S. stercoralis* larvae (12). Neutrophil elastase (NE) secreted following contact with *S. mansoni* is potentially toxic to a number of stages of this parasite (13).

Mast cells also play an important role in parasitic infections and have been implicated in the regulation of innate and adaptive immune responses following infection (14). Helminth infections are associated with elevations in tissue mast cell numbers (15). In the presence of helminth antigens, FcεRI receptor provokes mast cell degranulation, which results in the release of mast cell tryptase (MCT), carboxypeptidase-A3 (CPA-3), and leukotriene C4 (LTC4), which has direct cytotoxic effect on helminths (15, 16). During helminth infection, studies have revealed that mast cells are crucial in the expulsion of several helminth species from the gastrointestinal tract (17) including *T. spiralis, Nippostrongylus brasiliensis*, and *S. ratti* in rodent models (18, 19).

In this study, we wanted to characterize the presence and persistence of eosinophil, neutrophil, and mast cell degranulation proteins in *Ss* infection before and after treatment. We hypothesized that the plasma levels of granular proteins would reflect the activation profile of these important granulocyte subsets and its association to *Ss* infection. To this end, we measured the plasma levels of eosinophil granular proteins (ECP, EDN, EPX, and MBP), neutrophil granular proteins [NE, MPO, and proteinase-3 (PTN-3)], and mast cell granular proteins and mediators (MCT, LTC4, and CPA-3) in *Ss*-infected (INF) and *Ss*-uninfected (UN) individuals. Plasma levels of ECP, EPX, EDN, MBP, NE, MPO, MCT, LTC4, and CPA-3 levels were all significantly increased in *Ss* infection compared to those without *Ss* infection. These levels decreased significantly after anthelmintic treatment.

## Materials and Methods

### Ethics Statement

All participants were examined as a part of a natural history study protocol (12-I-073) approved by Institutional Review Boards of the National Institute of Allergy and Infectious Diseases (USA) and the National Institute for Research in Tuberculosis (India), and informed written consent was obtained from all participants.

### Study Population

We studied a total of 118 individuals including of 60 clinically asymptomatic, INF individuals and 58 UNF, endemic healthy individuals in Tamil Nadu, South India (Table 1). These individuals were all enrolled from a rural population. None had previous anthelmintic treatment, a history of helminth infections, or HIV. The INF individuals were followed up after 6 months of anthelmintic treatment.

**Table 1:** Baseline demographics of the study population.

Study demographics	Ss infected (INF)	Ss uninfected (UN)
Number	60	58
Gender (male/female)	33/27	35/23
Median age (range)	36 (20–65)	39 (20–60)
NIE ELISA	Positive	Negative

*Strongyloides stercoralis* infection was detected by measuring IgG antibodies to the recombinant NIE antigen, as explained elsewhere (20, 21). Further confirmation was done using specialized stool examination with nutrient agar plate cultures (22). None of the study population had lymphatic filariasis (based on ELISA) or other intestinal helminths (based on the stool microscopy). All INF individuals were treated with single doses of ivermectin and albendazole, and follow-up blood draws were collected after 6 months. Treated individuals were *Ss* infection negative by stool microscopy at 6 months posttreatment (post-T). All UN individuals were negative for anti-*Ss*-NIE and for filarial and other intestinal helminths.

### Measurement of Hematological Parameters

Hematological parameters were measured from fresh venous EDTA blood samples on all individuals using an ACT 5 Diff. hematology analyzer (Beckman Coulter, Brea, CA, USA).

### Measurement of Eosinophils, Neutrophils, and Mast Cell Granular Proteins

Plasma levels of ECP, EDN, EPX, MBP (MyBiosource, Inc., San Diego, CA, USA), MPO, PTN-3 (R&D Systems, Minneapolis, MN, USA), NE (Cell Sciences Hycult Biotech, Canton, MA, USA), MCT, LTC4, and CPA-3 were measured using the Mybiosource ELISA kits (MyBiosource, Inc., San Diego, CA, USA), followed the manufacturer’s protocol. The detection limits were as follows: ECP, 1.56–100 ng/ml; EDN, 0.625–40 ng/ml; EPX, 78–5,000 pg/ml; MBP, 0.468–30 ng/ml; MPO, 62.50–4,000 pg/ml; PTN-3, 15.6–1,000 pg/ml; NE, 0.4–25 ng/ml; MCT, 3.12–100 ng/ml; LTC4, 78–5,000 pg/ml; and CPA-3, 0.78–50 ng/ml. We have assigned the lowest standard value to the samples that were below the threshold of detection.

### Statistical Analysis

Data analyses were done using GraphPad PRISM 7 (GraphPad Software, Inc., San Diego, CA, USA). Central tendency was calculated using geometric mean (GM). Nonparametric Mann–Whitney *U* test and Wilcoxon matched pair test were used to calculate the statistical significant difference. Multiple comparisons were corrected using the Holm’s correction. JMP 13 (SAS) software was used to perform Spearman rank correlation matrix.

## Results

### *Ss* Infection Is Associated with Elevated Absolute Neutrophil and Eosinophil Counts and Reversal following Treatment

**Figure 1:**
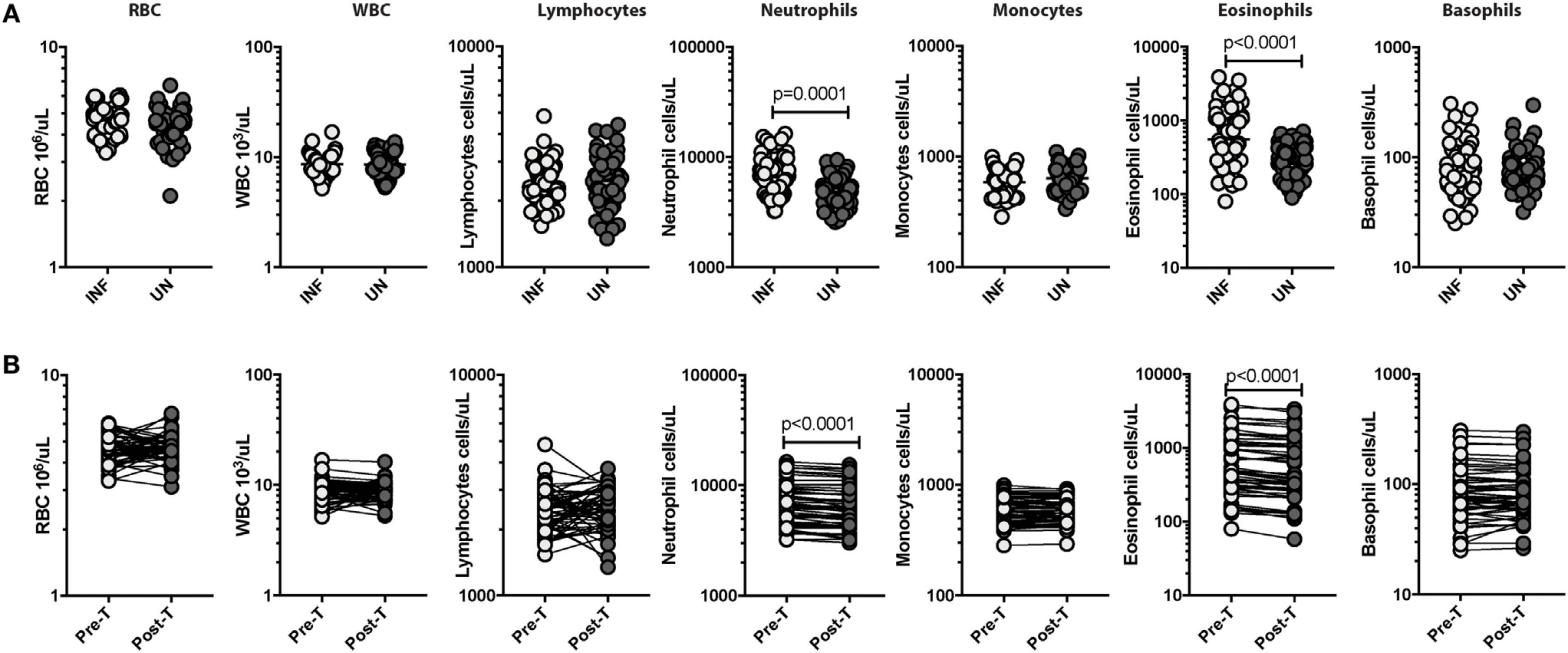
*Strongyloides stercoralis* (*Ss*) infection is associated with elevated absolute neutrophil and eosinophil counts and reversal following treatment. **(A)** Absolute counts of hematological parameters from Ss-infected (INF; *n* = 60) or uninfected (UN; *n* = 58) individuals were measured. Data are shown as scatter plots with the bar representing the geometric mean. *p* Values were calculated using the Mann–Whitney test with Holm’s correction for multiple comparisons. **(B)** Absolute counts of hematological parameters from Ss-infected pretreatment (pre-T; *n* = 60) and 6 months after treatment posttreatment (post-T) individuals were measured. *p* Values were calculated using the Wilcoxon matched pair test with Holm’s correction for multiple comparisons.

As shown in Table 1, there was no significant difference in age or gender between the two groups. We measured the hematological parameters in the two groups. As shown in Figure 1A, INF had significantly enhanced levels of neutrophils (GM of 6,566/μl in INF Vs GM of 4,848/μl in UN; *p* = 0.0001) and eosinophils (GM of 550/μl in INF vs GM of 281/μl in UN; *p* < 0.0001) in comparison with UN individuals. The other hematological parameters did not show any significant difference between the two groups. Upon anthelminthic treatment, the absolute numbers were significantly reversed. As shown in Figure 1B, absolute counts of neutrophils [GM of 6,566/μl in pretreatment (pre-T) vs GM of 6,000/μl in post-T; *p* < 0.0001] and eosinophils (GM of 550/μl in pre-T Vs GM of 483/μl in post-T; *p* < 0.0001) were significantly decreased. Other hematological parameters did not show any significant changes following treatment.

### *Ss* Infection Is Associated with Elevated Levels of Eosinophils, Neutrophils, and Mast Cell Granular Proteins

**Figure 2:**
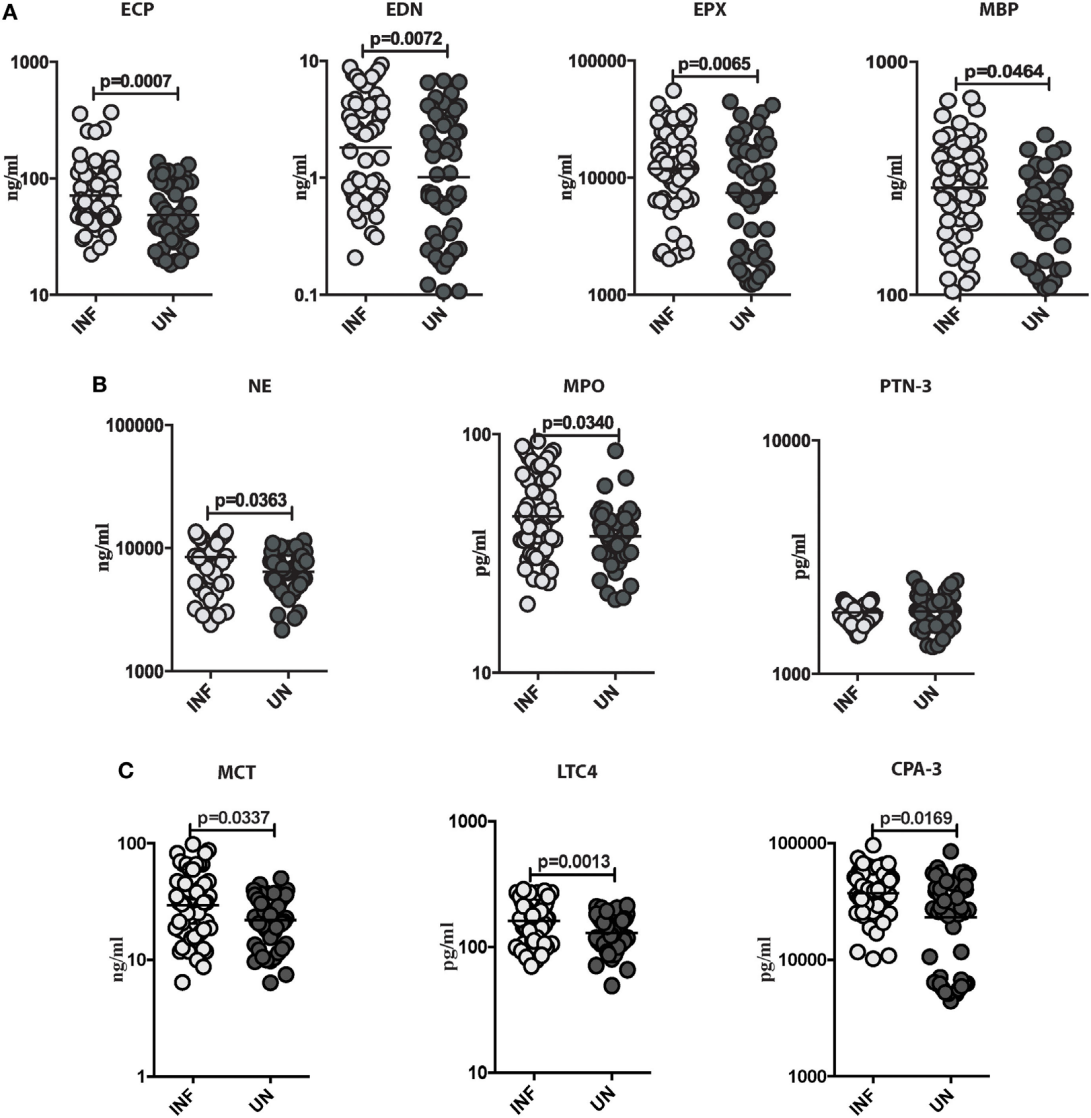
*Strongyloides stercoralis* (*Ss*) infection is associated with elevated levels of eosinophils, neutrophils, and mast cell granular proteins. **(A)** Plasma levels of eosinophil cationic protein (ECP), eosinophil-derived neurotoxin (EDN), eosinophil peroxidase (EPX), and major basic protein (MBP), from *Ss*-infected (INF; *n* = 60) or *Ss*-uninfected (UN; *n* = 58) individuals were measured by ELISA. Data are shown as scatter plots with the bar representing the geometric mean. *p* Values were calculated using the Mann–Whitney test. **(B)** Plasma levels of plasma levels of neutrophil elastase (NE), myeloperoxidase (MPO), and proteinase-3 (PTN-3) from (INF; *n* = 60) or (UN; *n* = 58) individuals were measured by ELISA. Data are shown as scatter plots with the bar representing the geometric mean. *p* Values were calculated using the Mann–Whitney test. **(C)** Plasma levels of plasma levels of mast cell tryptase (MCT), leukotriene C4 (LTC4), and carboxypeptidase A-3 (CPA-3) from INF (*n* = 60) or UN (*n* = 58) individuals were measured by ELISA. Data are shown as scatter plots with the bar representing the geometric mean. *p* Values were calculated using the Mann–Whitney test with Holm’s correction for multiple comparisons.

To characterize the role of eosinophils, neutrophils, mast cell granular proteins, and lipid mediators in *Ss* infection, we measured the plasma levels of eosinophil granular proteins (ECP, EDN, EPX, and MBP), neutrophil granular proteins (NE, MPO, and PTN-3), mast cell granular proteins, and lipid mediator (MCT, LTC4, and CPA-3) in INF and UN individuals. As shown in Figure 2A, INF had significantly higher levels of ECP (GM of 71.18 ng/ml in INF vs. 48.59 ng/ml in UN; *p* = 0.0007), EDN (GM of 1.811 ng/ml in INF vs. 1.014 ng/ml in UN; *p* = 0.0072), EPX (GM of 1,1945 ng/ml in INF vs. 7,407 ng/ml in UN; *p* = 0.0065), and MBP (GM of 288.4 ng/ml in INF vs. 223.7 ng/ml in UN; *p* = 0.0464) in comparison to UN individuals. As shown in Figure 2B, INF had significantly enhanced levels of NE (GM of 8,456 ng/ml in INF vs. 6,422 ng/ml in UN; *p* = 0.0363) and MPO (GM of 45.12 pg/ml in INF vs. 37.26 pg/ml in UN; *p* = 0.0340) in comparison to UN individuals. As shown in Figure 2C, INF had significantly increased levels of MCT (GM of 8,456 ng/ml in INF vs. 6,422 ng/ml in UN; *p* = 0.0363), LTC4 (GM of 45.12 pg/ml in INF vs. 37.26 pg/ml in UN; *p* = 0.0340), and CPA-3 (GM of 45.12 pg/ml in INF vs. 37.26 pg/ml in UN; *p* = 0.0340) in comparison to UN individuals.

### *Ss* Infection Is Associated with Decreased Levels of Eosinophils, Neutrophils, and Mast Cell Granular Proteins following Anthelminthic Treatment

**Figure 3:**
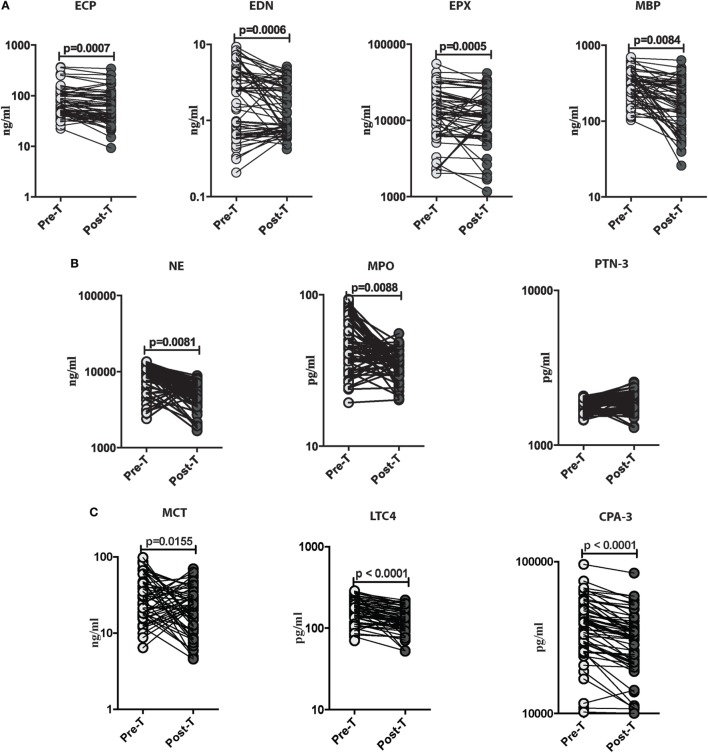
*Strongyloides stercoralis* (*Ss*) infection is associated with decreased levels of eosinophils, neutrophils, and mast cell granular proteins following anthelminthic treatment. **(A)** Plasma levels of eosinophil cationic protein (ECP), eosinophil-derived neurotoxin (EDN), eosinophil peroxidase (EPX), and major basic protein (MBP), from *Ss*-infected pre treatment (pre-T; *n* = 60) and 6 months following treatment from posttreatment (post-T) individuals were measured by ELISA. *p* Values were calculated using the Wilcoxon matched pair test. **(B)** Plasma levels of neutrophil elastase (NE), myeloperoxidase (MPO), and proteinase-3 (PTN-3) from *Ss* infected (pre-T; *n* = 60) and 6 months following treatment from post-T individuals were measured by ELISA. *p* Values were calculated using the Wilcoxon matched pair test. **(C)** Plasma levels of mast cell tryptase (MCT), leukotriene C4 (LTC4), and carboxypeptidase A3 (CPA-3) from *Ss*-infected (pre-T; *n* = 60) and 6 months following treatment from post-T individuals were measured by ELISA. *p* Values were calculated using the Wilcoxon matched pair test with Holm’s correction for multiple comparisons.

To determine the outcome of treatment on the levels of these granulocyte-associated proteins in those with *Ss* infection, all INF individuals were treated, and the levels of eosinophil granular proteins (ECP, EDN, EPX, and MBP), neutrophil granular proteins (NE, MPO, and PTN-3), and mast cell products (MCT, LTC4, and CPA-3) were measured in INF individuals before and after anthelminthic treatment. As shown in Figure 3A, the systemic levels of ECP (GM of 71.18 ng/ml in pre-T vs. 57.78 ng/ml in post-T; *p* = 0.0007), EDN (GM of 1.811 ng/ml in pre-T vs. 1.365 ng/ml in post-T; *p* = 0.0006), EPX (GM of 1,1945 ng/ml in pre-T vs. 1,1146 ng/ml in post-T; *p* = 0.0005), and MBP (GM of 288.4 ng/ml in pre-T vs. 167 ng/ml in post-T; *p* = 0.0084) were significantly decreased in INF individuals following anthelmintic treatment. For the neutrophil-associated proteins, as shown in Figure 3B, the systemic levels of NE (GM of 8,456 ng/ml in pre-T vs. 4,491 ng/ml in post-T; *p* = 0.0081) and MPO (GM of 45.12 pg/ml in pre-T vs. 36.58 pg/ml in post-T; *p* = 0.0088) were significantly decreased in INF individuals following anthelmintic treatment. Furthermore, the systemic levels of MCT (GM of 8,456 ng/ml in pre-T vs. 4,491 ng/ml in post-T; *p* = 0.0081), LTC4 (GM of 45.12 pg/ml in pre-T vs. 36.58 pg/ml in post-T; *p* = 0.0088), and CPA-3 (GM of 45.12 pg/ml in pre-T vs. 36.58 pg/ml in post-T; *p* = 0.0088) were also significantly diminished in INF individuals following anthelmintic treatment (Figure 3C).

### Relationship between Eosinophils, Neutrophils, and Mast Cell Granular Protein Levels and Absolute Numbers of Eosinophils, Neutrophils, and Basophils in INF Individuals

**Figure 4:**
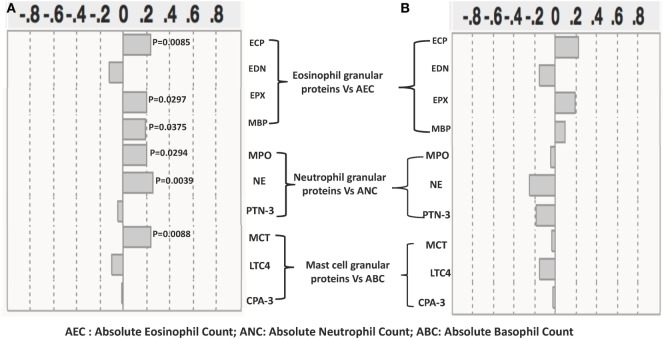
Relationship between eosinophil, neutrophil, and mast cell granular protein levels and absolute numbers of eosinophils, neutrophils, and basophils in *Strongyloides stercoralis* (*Ss*)-infected individuals and following anthelminthic treatment. **(A)** The absolute count of eosinophils was correlated with plasma levels of eosinophil cationic protein (ECP), eosinophil-derived neurotoxin (EDN), eosinophil peroxidase (EPX), and major basic protein (MBP); the absolute count of neutrophils was correlated with plasma levels of neutrophil elastase (NE), myeloperoxidase (MPO), and proteinase-3 (PTN-3), and the absolute count of neutrophils were correlated with plasma levels of mast cell tryptase (MCT), leukotriene C4 (LTC4), and carboxypeptidase A-3 in *Ss*-infected individuals (*n* = 60). **(B)** The absolute count of eosinophils correlation with plasma levels of ECP, EDN, EPX, and MBP; the absolute count of neutrophils correlation with plasma levels of NE, MPO, and PTN-3; and the absolute count of neutrophils correlation with plasma levels of MCT, LTC4, and carboxypeptidase A-3 (CPA-3) in *Ss*-infected following anthelmintic treated individuals (*n* = 60). *p* and *r* values were calculated using the Spearman rank correlation test using JMP software.

The relationships between the levels of eosinophils, neutrophils, and mast cell granular proteins and the absolute numbers of eosinophils, neutrophils, and basophils were next assessed (Figure 4A). There was a significant positive correlation between absolute eosinophil count (AEC) and the levels of ECP (*r* = 0.2413; *p* = 0.0085), EPX (*r* = 0.2196; *p* = 0.0169), and MBP (*r* = 0.1918; *p* = 0.0375). There was also a significant positive correlation between levels of NE (*r* = 0.2637; *p* = 0.0039) and MPO (*r* = 0.2006; *p* = 0.0294) and the absolute neutrophil count (ANC). Finally, there was also a significant positive correlation between the levels of MCT (*r* = 0.2637; *p* = 0.0039) and the absolute basophil count. Next, we assessed the correlation between the post anthelmintic treatment levels of eosinophils, neutrophils, and mast cell granular proteins and the absolute numbers of eosinophils, neutrophils, and basophils. We did not find any significant correlation between granular proteins and the absolute numbers of eosinophils, neutrophils, and basophils at the post-T time point (Figure 4B).

## Discussion

Eosinophils are one of the foremost components of the immune system, which play a prominent role in parasitic infections. Eosinophilia is a hallmark of helminth infections, and in some host–parasite interactions, eosinophils have been witnessed to kill worms and mediate protective immunity (6, 23, 24). Eosinophils are also presumed to play a role as APCs for the initiation of the primary and secondary Th2 immune responses to *S. stercoralis* (25), indicating an elemental role for eosinophils at the boundary between innate and adaptive immune responses. Eosinophils have secondary granules, which contain MBP, ECP, EDN, and EPO, and which are directly toxic to the larvae of *S. stercoralis* (26, 27).

Eosinophils and antibodies play a crucial function in defense mechanisms against *S. stercoralis* larvae in innate (28) and adaptive immune responses (29). Previous studies have shown that mice deficient in MBP and (30) and EPO (31) are more susceptible to Strongyloides infection. O’Connell et al. have shown that MBP involved in eosinophil-mediated larval killing (12). ECP and EDN possess ribonuclease activity that form pores into the membrane of target cells, facilitating the entry of other toxic molecules into the cells with subsequent degeneration (26). Plasma levels of eosinophil granule proteins deliver an indirect measure of degranulation in the tissues and are prominently augmented in many helminth-infected patients (32). Our data also show that eosinophil granular protein levels were increased in INF individuals. Earlier studies on onchocerciasis, lymphatic filarisis, schistosomiasis, and loiasis showed that ECP and EDN/EPX levels were elevated (32, 33). The serum concentrations of these proteins emerge consequently to reflect the functional activity of the corresponding granulocyte effector system in the host. In our study, we observed that there was a positive correlation between plasma levels of ECP, EPX and MBP, and the AEC, a finding similar to that seen in loiasis (32, 33). Thus, eosinophil granular proteins appear to reflect eosinophil activation.

Neutrophils are involved in the activation, regulation, and effector functions of innate and adaptive immune cells (34). NE and PTN-3 are directly involved in intracellular killing of phagocytosed bacteria in phagolysosomes, in conjunction with MPO and reactive oxygen species (35). During certain helminth infections, as with non-helminth induced inflammation, neutrophils are often the first cells to be recruited; these can mediate a degree of protective immunity against nematode parasites, as has been revealed most conclusively in the *Strongyloides sp*. model (9, 36, 37). Maximum killing happened by neutrophils when EPO from eosinophils attached to the surface of *S. mansoni* (38). In other mouse models, purified neutrophils have been shown to independently kill Strongyloides larvae (39). In addition, neutrophils are known to mediate adult worm killing through an MPO-dependent mechanism (12, 40). In our study, NE and MPO levels were significantly increased in INF individuals, and the levels were significantly associated with ANCs. This is similar to an earlier study on *Onchocerca volvulus* infection that showed that the plasma level of MPO was correlated with ANC (32, 41). Changes in the PTN-3 levels may be due to increased production during inflammatory activity and neutrophil or mononuclear cell leakage/degranulation. PTN-3 has antimicrobial properties and is known to efficiently kill bacteria (41). However, in our study, PTN-3 did not show any significant alterations in Ss infection. Thus, neutrophil granular proteins, similar to their eosinophil counterparts, appear to reflect neutrophil-mediated activation in *Ss* infection.

Mast cell tryptase, CPA-3, and arachidonic acid-derived lipid mediators such as LTC4 are produced during mast cell activation (42, 43). MCT is a major protein product of human mast cells (44). During the activation of mast cells, MCT levels have been shown to be elevated in anaphylaxis (45) and systemic mastocytosis (46). Infection with *T. spiralis* has shown increased numbers of gastrointestinal tract mast cells and associated levels of LTC4, that was felt to be involved in rapid worm expulsion (47). In line with these data, INF individuals showed significantly increased levels of MCT, CPA-3, and LTC4 when compared with UN individuals.

Earlier studies have demonstrated that eosinophils and neutrophils are the key players mediating microfilarial killing following anthelmintic treatment (48). Destruction of parasites occurs through eosinophil degranulation after anthelmintic treatment with DEC or ivermectin (49, 50). Cooper et al. showed that plasma levels of MCT increased following treatment for onchocerciasis (51). In this study, we show that the augmented levels of eosinophils, neutrophils, and mast cell granular proteins are significantly diminished at 6 months following treatment. This indicates that the release of these granular proteins is intimately related to the presence of active helminth infection and that elimination of the parasite removes the stimulus for increased release of these factors.

Our study adds to the growing body of literature showing the importance of granulocytes and their activation in helminth infections. While the roles of neutrophils in animal models of helminth infections are well studied (36), very scant data exist on the role of these important innate mediators in human helminth infection. Thus, our study derives strength from the fairly large sample size and the homogeneity of the population studied. Further studies exploring the exact role of these granular proteins should provide valuable insight into the regulation of the protective or pathogenic immune response in helminth infections at large.

## Ethics Statement

All individuals were examined as part of a natural history study protocol approved by Institutional Review Boards of the National Institute of Allergy and Infectious Diseases (USA) and the National Institute for Research in Tuberculosis (India), and informed written consent was obtained from all participants.

## Author Contributions

Conceived and designed the experiments: AR and SB. Performed the experiments: AR, SM, and YB. Analyzed the data: AR and SB. Contributed reagents/materials/analysis tools: CD and TN. Wrote the paper: AR, TN, and SB.

## Conflict of Interest Statement

The authors declare that the research was conducted in the absence of any commercial or financial relationships that could be construed as a potential conflict of interest. The reviewer WK and handling Editor declared their shared affiliation.
